# Application of Diffusion Kurtosis Imaging in Evaluating Acute Xerostomia in Nasopharyngeal Carcinoma Treated With Induction Chemotherapy Plus Concurrent Chemoradiotherapy

**DOI:** 10.3389/fonc.2022.870315

**Published:** 2022-05-19

**Authors:** Da-Wei Zhao, Xue-Mei Fang, Shu-Han Zhou, Yan-Rong Luo, Jian Wei, Kun Liu, Ling-Ling Meng, Gang Liu, Jin-Feng Li, Xiao Zang, Meng Li, Xiao-Fei Qiang, Hua-Yong Jiang, Wen-jun Fan, Xin-Xin Zhang, Lin Ma

**Affiliations:** ^1^ Medical School of Chinese PLA, Beijing, China; ^2^ Department of Radiology, Characteristic Medical Center of Chinese People’s Armed Police Force, Tianjin, China; ^3^ Department of Radiation Oncology, First Medical Center of PLA General Hospital, Beijing, China; ^4^ Department of Ultrasound, Tianjin Medical University General Hospital Airport Hospital, Tianjin, China; ^5^ Department of Radiation Oncology, Sun Yat-sen University Cancer Center, State Key Laboratory of Oncology in South China, Collaborative Innovation Center for Cancer Medicine, Guangdong Key Laboratory of Nasopharyngeal Carcinoma Diagnosis and Therapy, Guangzhou, China; ^6^ Department of Otolaryngology, First Medical Center of PLA General Hospital, Beijing, China; ^7^ Department of Radiology, First Medical Center of PLA General Hospital, Beijing, China; ^8^ Department of Radiology, Helicopter Detachment of the Second Mobile Corps of the Armed Police, Chengdu, China; ^9^ Department of Radiation Oncology, Fifth Medical Center of the PLA General Hospital, Beijing, China; ^10^ Affiliated Foshan Maternity & Child Healthcare Hospital, Southern Medical University, Foshan, China; ^11^ Armed Police Forces Corps Hospital of Henan Province, Zhengzhou, China

**Keywords:** xerostomia, diffusion kurtosis imaging, parotid gland, submandibular gland, sublingual gland, radiation

## Abstract

**Purpose:**

The aim of this study was to identify the efficacy of diffusion kurtosis imaging (DKI) in tracking and monitoring the dynamic change of parotid glands (PGs), submandibular glands (SMGs), sublingual glands (SLGs), and acute xerostomia in nasopharyngeal carcinoma (NPC) patients treated with induction chemotherapy (IC) plus concurrent chemoradiotherapy (CCRT).

**Methods:**

The prospective study recruited 42 participants treated with IC+CCRT. All patients underwent DKI scanning six times: before IC, before RT, in the middle of the RT course, immediately after RT, and 1 and 3 months post-RT. Mean diffusion coefficient (MD) and mean kurtosis (MK) of PG, SMG, SLG, saliva flow rate measured under resting (uSFR) and stimulated condition (sSFR), and xerostomia questionnaire (XQ) scores were recorded.

**Results:**

At each time point, sSFR was significantly higher than uSFR (*p* < 0.05 for all). MD of the salivary glands and XQ scores increased over time while MK, uSFR, and sSFR decreased. After IC, the significant differences were detected in MD and MK of bilateral SMG and MK of the left SLG (*p* < 0.05 for all), but not in MD and MK of PG, uSFR, sSFR, and XQ scores. After RT, sSFR at 1m-RT decreased significantly (*p* = 0.03) while no significant differences were detected in uSFR and XQ scores. Moderate-strong correlations were detected in ΔMD-PG-R%, ΔMK-PG-R%, ΔMD-PG-L%, ΔMK-PG-L%, ΔMD-SMG-R%, ΔMK-SMG-R%, ΔMD-SMG-L%, ΔMK-SMG-L%, and ΔMD-SLG-R%, with correlation coefficients (*p* < 0.05 for all) ranging from 0.401 to 0.714. ΔuSFR% was correlated with ΔMD-SMG% (*p* = 0.01, *r* = −0.39), ΔMD-SLG% (*p* < 0.001, *r* = −0.532), and ΔMK-SMG% (*p* < 0.001, *r* = −0.493). ΔsSFR% correlated with ΔMD-PG% (*p* = 0.001, *r* = −0.509), ΔMD-SMG% (*p* = 0.015, *r* = −0.221), and ΔMK-PG% (*p* < 0.001, *r* = 0.524). ΔXQ% was only correlated with ΔMK-PG% (*p* = 0.004, *r* = 0.433).

**Conclusion:**

DKI is a promising tool for tracking and monitoring the acute damage of PG, SMG, and SLG induced by IC+CCRT in NPC patients.

## Introduction

The widespread application of intensity-modulated radiotherapy (RT) and optimization of chemotherapy strategies have contributed to improved survival with reduced toxicities in nasopharyngeal carcinoma (NPC) (1). As NPC is highly sensitive to ionizing radiation, RT has been regarded as the mainstay treatment modality (1, 2). Besides, induction chemotherapy (IC) is beneficial for eradicating micro-metastases earlier and reducing tumor size before RT to improve protecting organs at risks (3). Therefore, IC followed by concurrent chemoradiotherapy (CCRT) may represent a promising treatment strategy for NPC (1, 4, 5).

Since RT targets overlap residence of the salivary glands, apoptosis of acinar cells of irradiated glands inevitably leads to ablation of saliva output and xerostomia (6), a prevalent and long-lasting adverse effect of RT (2, 7–9). Acute xerostomia within 3 months after RT was the most serious and the most difficult period (10) for patients because of the limited efficacy of treatment (2). Furthermore, moderate-dose chemotherapy is thought to be another cause of developing xerostomia in breast cancer, although the loss of function tends to be milder and less long lasting (11–13). Nevertheless, there is no essential evidence in the literature on the causal association between salivary glands’ hypofunction and chemotherapy in NPC.

The major salivary glands, including parotid (PG), submandibular (SMG), and sublingual glands (SLG), are in charge of more than 90% saliva production (9, 14). PG produces approximately 50% of the total volume of stimulated whole saliva while the majority of saliva is secreted by SMG and SLG under resting conditions (9). Limiting radiation dose of the major glands through intensity-modulated RT (IMRT) and sparing SMG techniques have demonstrated a reduction in salivary gland hypofunction and xerostomia (6, 15, 16).

As the reference standard, salivary gland scintigraphy can provide functional information of irradiated glands (17), but it is unsuitable for clinical practice due to its invasive nature and additional radiation exposure (18). Saliva flow rate (SFR) measurement and Xerostomia Questionnaire (XQ) were prevalent in evaluating xerostomia in clinical settings despite the low reproducibility and non-specificity (6, 10, 19). Diffusion kurtosis imaging (DKI), an advanced diffusion technique based on non-Gaussian diffusion distribution assumption, is of capacity in reflecting the true water diffusion *in vivo* (20). In recent years, the application of DKI indeed provide an opportunity to get further insights into the microstructure change of irradiated salivary glands without invasiveness (21). However, the techniques merely focused on investigating PG injury, and there had been no study on evaluating SMG and SLG by DKI ever before.

The primary objective of the prospective study was to verify the efficacy of DKI in tracking and monitoring the change of PG, SMG, and SLG in NPC patients treated with IC+CCRT. We investigated the differences in MD and MK between the left and right salivary glands and compared ipsilateral PG, SMG, and SLG at baseline. The dynamic change in DKI parameters, uSFR, sSFR, and XQ scores for all the major salivary glands was also detected by pairwise comparisons between six time points from before IC to 3 months after RT.

## Materials and Methods

### Patients

Fifty NPC patients who met eligibility criteria who were scheduled to receive radical RT at our institution were recruited from January to September 2020. All patients have been diagnosed with NPC pathologically; had a good performance status (KPS ≥ 70 or ECOG 0–1); were candidates for MRI examination in our clinics; had no distant metastasis, RT, or surgery to the head and neck region; and had no salivary gland diseases or any other medical causes of xerostomia. Patients with contraindications to chemoradiotherapy or whose MR images had artifacts constrained further analysis were excluded. This study was approved by the ethics board of the Chinese PLA General Hospital, and registered on July 6, 2019, in the Chinese Clinical Trial Registry (ChiCTR1900024328) (http://www.chictr.org.cn/showproj.aspx?proj=40726). Written informed consents were obtained before enrollment.

### Treatment

Two cycles of IC (docetaxel: 70 mg/m^2^ on day 1; cisplatin 40 mg/m^2^ on days 1 and 2) and three cycles of concurrent chemotherapy (cisplatin 70 mg/m^2^ or docetaxel 70 mg/m^2^) were administrated to all patients at 3-week intervals.

Helical tomotherapy (Hi-Art Tomotherapy; Accuray Inc., Sunnyvale, CA) was applied to all patients with a 6-MV photon beam. After the thermoplastic head and neck mask was used for immobilization, patients underwent enhanced CT scans with 3-mm slice thickness. Pinnacle 3.8.0 treatment workstation (Philips Medical Systems, Fitchburg, WI, USA) was used for target delineation and treatment planning optimization. Radiation targets typically included bilateral neck levels II, III, and IV, and level IB would be included in the high-risk clinical target volume (CTV1) in case of level IIA involvement, judged by clinicians based on evidence of histopathology and radiologic imaging. CTV was expanded uniformly by 3 mm to produce planning target volumes (PTV). The total prescribed dose for gross target volumes of the primary tumor (pGTVnx) and metastatic lymph node (pGTVnd) was 67.5 Gy for 30 fractions, while high-risk planning target volume (PTV1) was given 60 Gy (2.0 Gy per fraction) and low-risk planning target volume (PTV2) was given 54 Gy (1.8 Gy per fraction). The intensity-modulated planning system and SMG-sparing technique were applied to constrain radiation doses to ipsilateral SMG and SLG, while ensuring that the coverage of targets was more than 97%. The mean dose constraints for the spared SMG were 28 Gy; more information about plan optimization and dose-volume constraints for organs at risk were detailed in a previous publication (19). RT was delivered over one faction daily, five fractions weekly. Daily image-guided RT was implemented to verify setup before each faction. Neither salivary gland stimulators nor protectors were permitted.

### MR Scan Protocol

All patients were scheduled for six MRI examinations with the same scan protocols: before IC, before RT, mid-RT (in the middle of the RT course), post-RT (immediately after RT), 1 month (1m-RT), and 3 months (3m-RT) post-RT. All MRI examinations were performed on a 3.0-T MR scanner (Signa HDx, GE Healthcare, Milwaukee, WI, USA). Conventional MRI sequences, including axial, sagittal, and coronal T2-weighted 2D turbo spin-echo images, were obtained with a 16-channel neurovascular head and neck array coil. The DKI sequence was performed using a single-shot spin echo-planar imaging sequence with fast suppression (TR = 3,500 ms, TE = 86.8 ms, slice thickness = 6.0, slice gap = 1.0, bandwidth = 250.0, *b* values = 1, 500, 1,000, and 2,000 mm^2^/s). The diffusion gradients were applied in three orthogonal gradient diffusion directions; the images range from the skull base to the level of the glottis. The DKI acquisition time was 4:09.

### Data Analysis

The DKI parameter maps were obtained using the Functool software (Advanced Workstation version 4.6, GE Healthcare). The DKI model yielded two variables while S0 is known, according to the following equation: $S_{i}=S_{0}\times \exp\left(-b_{i}\times D+\frac{1}{6}bi^{2}\times D^{2}\times K\right)$ (20). with *S*_0_, *D*, and *K* as fitting variables, where *S_i_* is the signal at a particular *b* value and *S*_0_ is the baseline signal without diffusion gradient. Accordingly, *D* is diffusivity and *K* describes peakedness of a probability of water distribution. The parameter MD is the mean diffusion coefficient in normal diffusion after correcting the non-Gaussian effect, while MK is the mean kurtosis reflecting non-Gaussian diffusion behavior.

**Figure 1:**
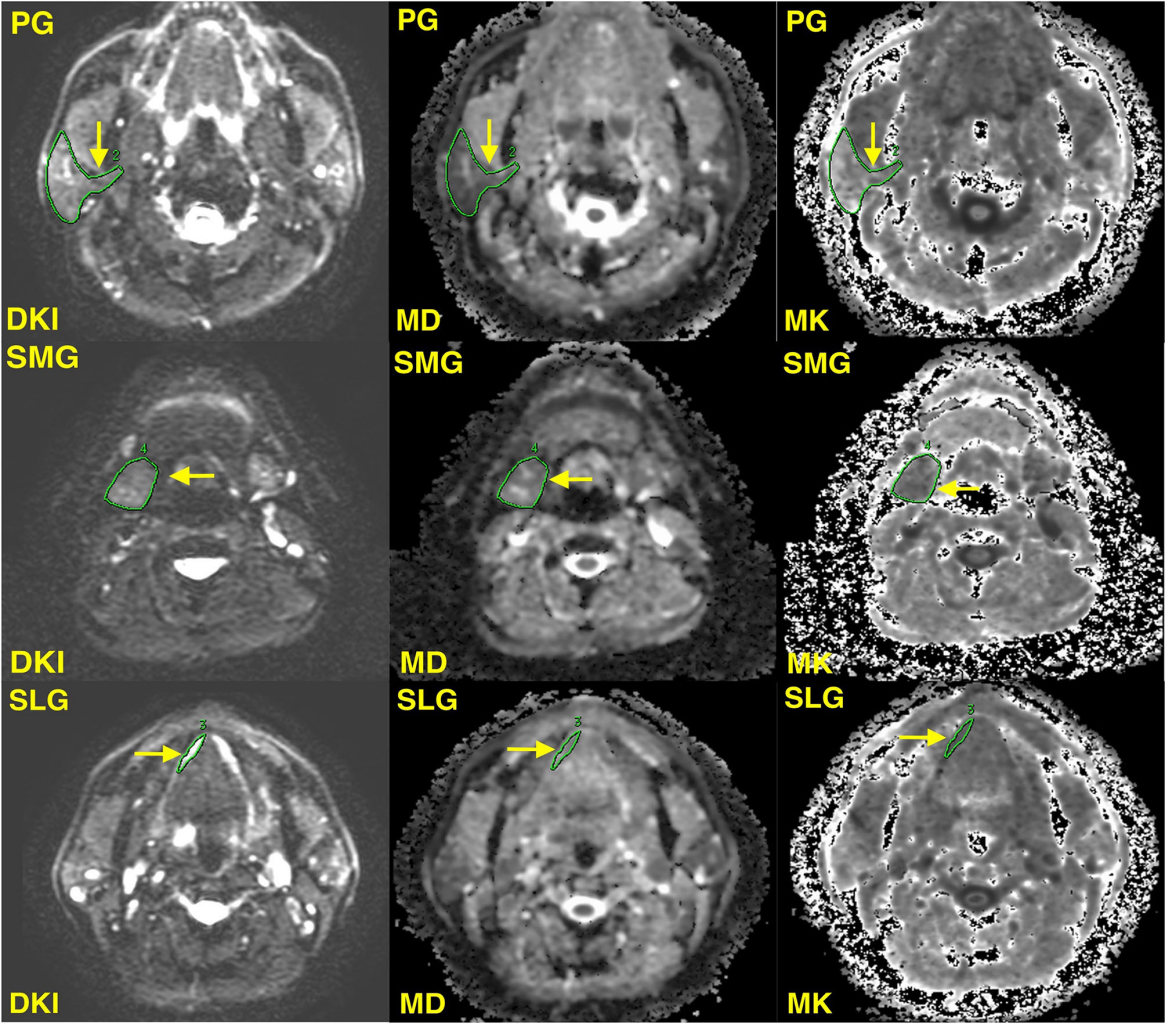
Illustrations of the region of interest of the right parotid gland (PG), sublingual gland (SLG), and submandibular gland (SMG) (yellow solid arrow). DKI, diffusion kurtosis imaging; MD, mean diffusion; MK, mean kurtosis.

Two radiologists who had at least 8 years of experience in head and neck MR imaging independently analyzed MR images blind to clinical data. Taking axial T2 images as a reference, regions of interest (ROIs) were manually drawn on three slices of DKI parameter maps from the upper, middle, and lower levels of bilateral PG, SMG, and SLG (less than three slices of ROIs were acceptable for SLG because of its small volume constraints) to encompass as much of the gland parenchyma as possible. To reduce measurement inaccuracy, the major vessels in the glands were instructed to be excluded (**Figure 1**). The average of MD and MK values of three slices was recorded as the value of every single salivary gland.

### XQ Scores and SFR Measurement

All participants were instructed to complete the patient-reported xerostomia questionnaire (XQ) at each follow-up time point. The modified XQ was based on a version of the University of Michigan that has been validated and applied for years in the clinic (6, 10, 19). Briefly, the XQ was composed of 10 items in total, 5 items are associated with eating, speaking, swallowing, and chewing while the remaining 5 items are about the feeling of xerostomia at rest. Each response is scored on a four-point scale ranging from 0 to 3, with higher scores indicating more severe xerostomia. The results of ten questions were added together to get a summary score ranging from 0 to 30.

SFR measurements were taken at each follow-up time point before the MRI scan. Patients were requested to spit saliva into a graded tube for 5 min under unstimulated settings, then repeat the procedure while salivary glands were stimulated by dipping a cotton bud into 2% citric acid dipped on the tongue tip once every 20 s. The volume of saliva collected was computed and recorded as SFR under unstimulated (uSFR) and stimulated conditions (sSFR) circumstances, respectively.

The change ratio of MD and MK of the salivary glands, uSFR, sSFR, and XQ scores from pre-RT to post-RT was calculated as the following equation:


Δ(parameters)%=(post(parameters)−pre(parameters))/pre(parameters)%

### Statistical Analysis

Statistical analyses were performed in R software (version 4.1.0; http://www.r-project.org). “multcomp”, “ggpubr”, “ggplot2”, “patchwork”, “pheatmap”, and “psych” packages were used for analysis. At baseline, paired *t*-test or Wilcoxon signed-rank test (according to the normality of data distributed) was selected to compare MD and MK of bilateral salivary glands at each time point. Dynamic changes in MD and MK of the salivary glands, uSFR, sSFR, and XQ scores during the follow-up period were analyzed by Friedman test or Kruskal–Wallis test. The correlation analyses were performed using Pearson correlation. Unlike the one-to-one correspondence that exists between the dose of bilateral salivary glands with the change ratio of DKI metrics of bilateral salivary glands. The damage of both the left and right salivary glands as a whole contributes to the change in uSFR, sSFR, and XQ scores. Hence, the final metrics’ value of the salivary gland was calculated by averaging the values of the left and right glands to analyze correlation coefficients between the change ratio of DKI metrics with uSFR, sSFR, and XQ scores. The intra-observer reproducibility of MD/MK of bilateral PGs, SMGs, and SLGs were analyzed using the intraclass correlation coefficient (ICC). Two-sided *p*-values < 0.05 were considered statistically significant.

## Results

Eight of 50 NPC patients were excluded (3 patients failed to complete all MRI scans, 1 patient due to bone metastasis, 2 withdrew informed consent, and the remaining 2 had poor-quality images). The highest T and N stage was T2 in 50.4% of patients and N2 in 42.8% of patients. Non-cornification undifferentiated subtypes accounted for 50% of NPC patients. The mean dose of bilateral SMGs was the highest among the major salivary glands: 50.20 Gy (22.82–64.77) and 47.89 Gy (25.46–66.45), respectively (demographic and clinical characteristics are summarized in **Table 1**).

**Table 1:** Demographic and clinical characteristics of patients.

Characteristics	Value
**Age**	43.19 ± 12.12
**Gender**	
Male	33 (78.6%)
Female	9 (21.4%)
**AJCC T stage**	
T1	2 (4.8%)
T2	22 (50.4%)
T3	13 (30.9%)
T4	5 (11.9%)
**AJCC N stage**	
N0	1 (2.4%)
N1	7 (16.7%)
N2	18 (42.8%)
N3	16 (38.1%)
**Pathological classification**	
Non-cornification undifferentiated	21 (50%)
Low differentiated	17 (40.5%)
Moderately differentiated	4 (9.5%)
**Mean dose of bilateral salivary glands**	
PG-R	32.58 Gy (26.06–41.44)
PG-L	33.01 Gy (27.34–42.56)
SMG-R	50.20 Gy (22.82–64.77)
SMG-L	47.89 Gy (25.46–66.45)
SLG-R	31.35 Gy (17.46–57.66)
SLG-L	28.39 Gy (17.17–45.81)

PG-R, right parotid gland; PG-L, left parotid gland; SMG-R, right submandibular gland; SMG-L, left submandibular gland; SLG-R, right sublingual gland; SLG-L, left sublingual gland. AJCC 8th edition (AJCC-8th) staging system was used to determine patients’ AJCC stage.

Intra-reproducibility of MD values of the major salivary glands was excellent, and ICCs of PG-R, PG-L, SMG-R, SMG-L, SLG-R, and SLG-L were 0.88, 0.91, 0.87, 0.92, 0.84, and 0.85 respectively. As for MK values, ICCs were 0.87, 0.86, 0.87, 0.86, 0.89, and 0.83. The metrics’ values of glands were recorded as the mean of two radiologists’ measurements.

### Comparison of MD/MK of the Salivary Glands at Baseline

**Figure 2:**
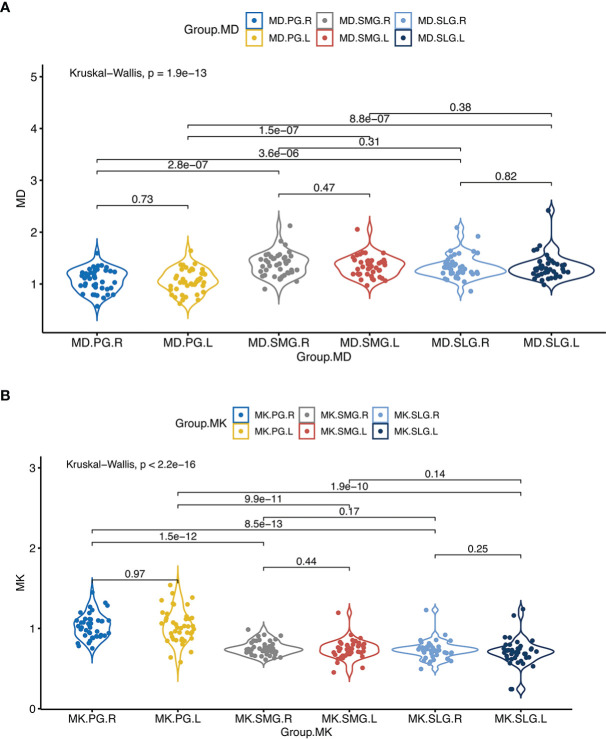
**(A)** denotes comparisons of MD between the left and the right side of the major salivary glands, and between ipsilateral PG, SMG, and SLG; **(B)** denotes comparisons of MK between the left and the right side of the major salivary glands, and between ipsilateral PG, SMG, and SLG. Abbreviations are referred to in **Table 2**.

There were no significant differences in MD and MK between the left and right salivary glands (*p* > 0.05 for all). Compared with ipsilateral SMG, MK of PG was significantly higher while MD was lower (*p* < 0.001 for all). Compared with ipsilateral SLG, MK of PG was significantly higher, while MD was lower (*p* < 0.001 for all). However, there were no significant differences between MD and MK of SLG and SMG (**Table 2**, **Figure 2**).

**Table 2:** MD and MK of bilateral PGs, SMGs, and SLGs with uSFR, sSFR, and XQ scores at each time point.

	MD (m ± sd)						MK (m ± sd)						SFR (m ± sd)		XQ		
	PG		SMG		SLG		PG		SMG		SLG		uSFR	sSFR		
	R	L	R	L	R	L	R	L	R	L	R	L					
**t1**	1.07 ± 0.22^bc^	1.06 ± 0.22^bc^	1.37 ± 0.23	1.34 ± 0.21	1.33 ± 0.23	1.33 ± 0.24	1.03 ± 0.15b^c^	1.03 ± 0.21^bc^	0.76 ± 0.09	0.74 ± 0.12	0.73 ± 0.12	0.71 ± 0.17	1.53 ± 2.24^e^	3.76 ± 2.73	4.55 ± 3.48		
**t2**	1.11 ± 0.22^bc^	1.09 ± 0.24^bc^	1.54 ± 0.24	1.50 ± 0.21	1.46 ± 0.26	1.42 ± 0.26	0.99 ± 0.16^bc^	1.04 ± 0.20^bc^	0.71 ± 0.07	0.70 ± 0.06	0.69 ± 0.10	0.67 ± 0.12	1.48 ± 1.41^e^	3.12 ± 2.41	5.21 ± 3.65		
**t3**	1.38 ± 0.31^c^	1.38 ± 0.31^bc^	1.70 ± 0.31^d^	1.65 ± 0.26^d^	1.57 ± 0.29	1.55 ± 0.29	0.90 ± 0.18^bc^	0.90 ± 0.18^bc^	0.67 ± 0.10	0.66 ± 0.09	0.66 ± 0.17	0.65 ± 0.18	0.55 ± 0.58^e^	1.16 ± 1.37	16.31 ± 4.14		
**t4**	1.56 ± 0.32^c^	1.55 ± 0.33^b^	1.94 ± 0.33^d^	1.89 ± 0.30^d^	1.74 ± 0.27	1.68 ± 0.30	0.83 ± 0.17^bc^	0.82 ± 0.17^bc^	0.60 ± 0.06d	0.59 ± 0.06d	0.56 ± 0.09	0.55 ± 0.07	0.22 ± 0.42^e^	0.49 ± 0.72	18.60 ± 3.91		
**t5**	1.66 ± 0.26^bc^	1.63 ± 0.28^c^	1.99 ± 0.28^a^	1.93 ± 0.29	1.80 ± 0.26	1.73 ± 0.30	0.75 ± 0.15^abc^	0.77 ± 0.15^bc^	0.59 ± 0.08d	0.60 ± 0.08d	0.56 ± 0.08	0.56 ± 0.08	0.12 ± 0.25^e^	0.28 ± 0.41	17.12 ± 5.64		
**t6**	1.70 ± 0.26^bc^	1.68 ± 0.24^b^	2.05 ± 0.24^d^	1.99 ± 0.32^d^	1.85 ± 0.27^a^	1.75 ± 0.34	0.74 ± 0.14^bc^	0.74 ± 0.14^bc^	0.57 ± 0.06	0.57 ± 0.07	0.58 ± 0.10	0.57 ± 0.10	0.07 ± 0.11^e^	0.17 ± 0.17	16.60 ± 5.15		

MD, mean diffusion; MK, mean kurtosis. PG, parotid gland; SMG, submandibular gland; SLG, sublingual gland; R, right; L, left; t1, prior to induction chemotherapy; t2, before radiotherapy; t3, middle of radiotherapy; t4, immediately after radiotherapy; t5, 1 month after radiotherapy; t6, 3 months after radiotherapy. ^a^denotes a significant difference between the left and right salivary glands. ^b^denotes a significant difference between ipsilateral PG and SMG. ^c^denotes a significant difference between ipsilateral PG and SLG. ^d^denotes a significant difference between ipsilateral SMG and SLG. ^e^denotes a significant difference between uSFR and sSFR.

### The Dynamic Change of MD and MK of the Salivary Glands, uSFR, sSFR, and XQ Scores

**Figure 3:**
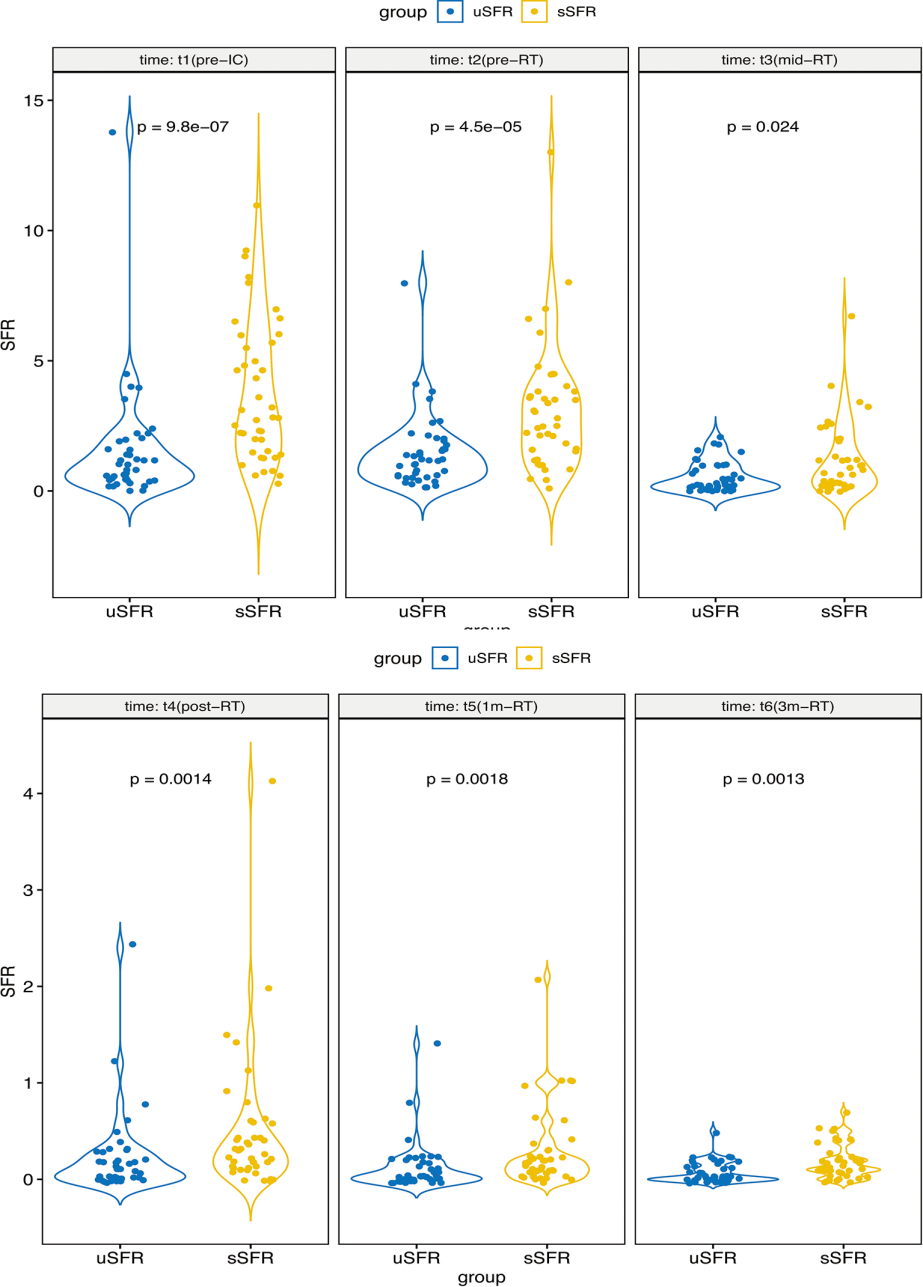
The comparisons of uSFR and sSFR at pre-IC, pre-RT, mid-RT, post-RT, 1m-RT, and 3m-RT denote that sSFR was significantly higher than uSFR at each time point. Abbreviations are referred to in **Table 2**.

At each time point, sSFR was significantly higher than uSFR (*p* < 0.001, *p* < 0.001, *p* = 0.024, *p* = 0.001, *p =* 0.002, *p* = 0.001, respectively) (**Figure 3**). MD of PG, SMG, SLG, and XQ scores increased over time, while MK, uSFR, and sSFR decreased significantly.

Compared with pre-IC, MD and MK of bilateral PG at post-RT showed no significant differences. Significant changes were found in MD and MK of bilateral SMG and SLG between the time point of pre-IC and pre-RT, except for MK of the right SLG (*p* = 0.078) and MK of the left SLG (*p* = 0.16). In addition, no significant difference was found in uSFR, sSFR, and XQ scores before and after IC.

Compared with pre-RT, MD of bilateral PG, SMG, and SLG at mid-RT and post-RT was significantly increased while MK decreased (*p* < 0.05 for all). Significant differences were also detected in MD and MK of all salivary glands except for MK of the right SLG (*p* = 0.08) between mid-RT and post-RT. There were also significant differences for uSFR, sSFR, and XQ scores between the time points of pre-RT, mid-RT, and post-RT (*p* < 0.05 for all).

**Figure 4:**
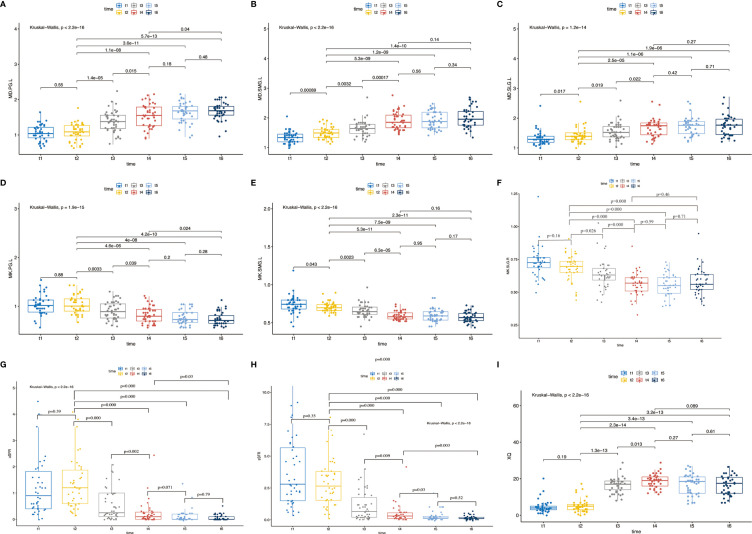
**(A–I)** The pairwise comparisons of MD and MK of left PG, SMG, SLG, and uSFR, sSFR, and XQ between different time points; *p* < 0.05 denotes significant differences between two time points linked together. Abbreviations are referred to in **Table 2**.

Compared with post-RT (t4), no significant differences were detected in MD and MK of the bilateral PG, SMG, and SLG except for MK-PG-R (*p* = 0.032) at 1m-RT. There were no significant differences between 1m-RT and 3m-RT for MD and MK of all salivary glands. Compared with post-RT, the trend of a decrease in uSFR was observed at 1m-RT but did not reach statistical significance (*p* = 0.071), while a significant decrease was found in sSFR (*p* = 0.03). XQ scores between time points of post-RT and 1m-RT showed no significant differences. There were also no significant differences between 1m-RT and 3m-RT in uSFR, sSFR, and XQ (**Table 2**, **Figure 4**, and **Supplementary Material**).

### Correlations Between the Change Ratio of DKI Metrics From Pre-RT to Post-RT With Dose of Salivary Glands

**Figure 5:**
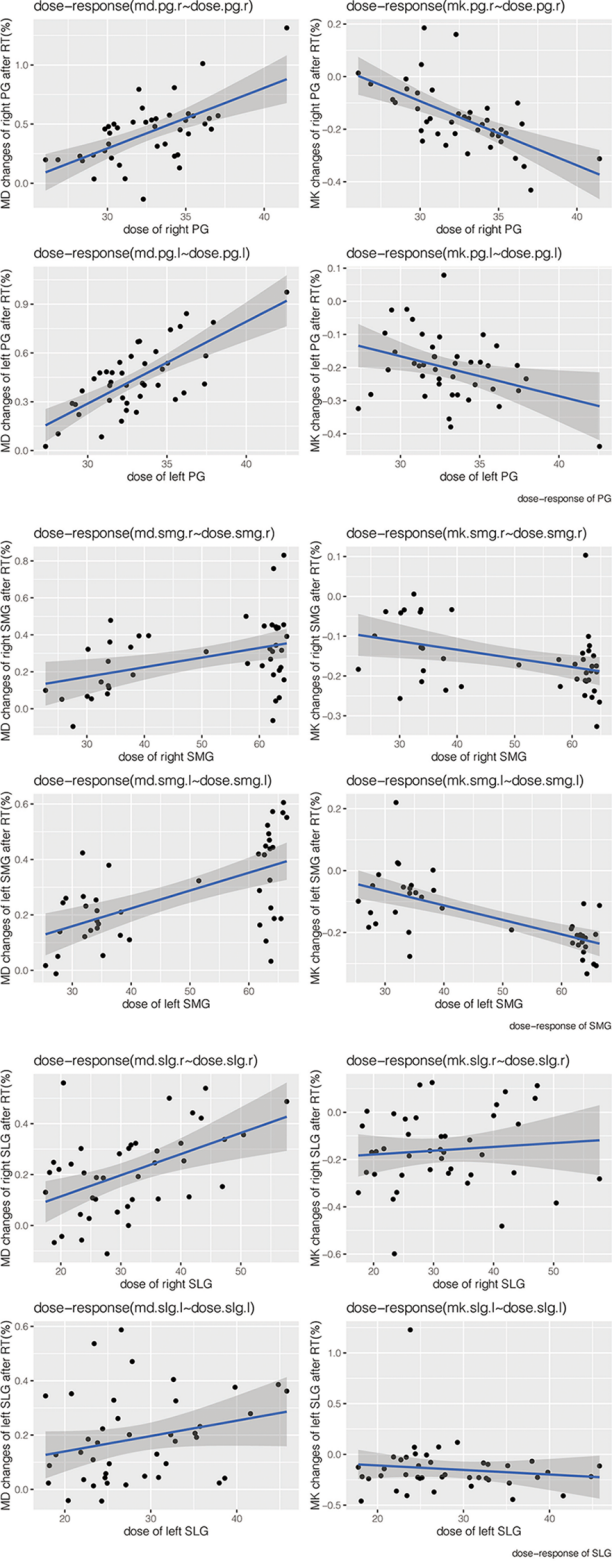
Scatter dot plots of the change ratio of MD and MK versus dose of bilateral PGs, SMGs, and SLGs. Abbreviations are referred to in **Table 2**.

**Figure 6:**
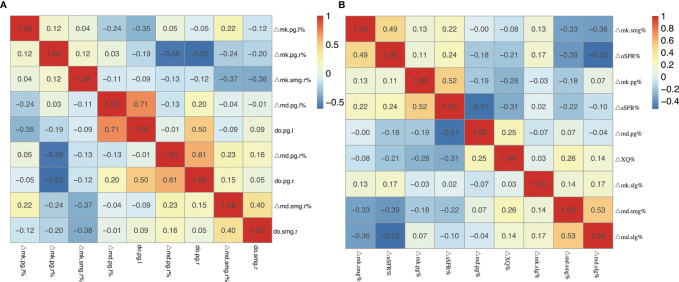
**(A)** Heatmap of the correlation coefficients between the change ratio of DKI metrics (MD and MK) from pre-RT to post-RT and dose of bilateral salivary glands. **(B)** Heatmap of the correlation coefficients between the change ratio of DKI metrics (MD and MK) of the salivary glands and the change ratio of uSFR, sSFR, and XQ scores from pre-RT to post-RT. Abbreviations are referred to in **Table 2**.

The change ratio of MD increased while the change ratio of MK decreased with the increasing dose of bilateral PG, SMG, and SLG (except for MK of bilateral SLG) (**Figure 5**). Moderate-strong correlations were detected in ΔMD-PG-R%, ΔMK-PG-R%, ΔMD-PG-L%, ΔMK-PG-L%, ΔMD-SMG-R%, ΔMK-SMG-R%, ΔMD-SMG-L%, ΔMK-SMG-L%, and ΔMD-SLG-R%, with correlation coefficients (*p* < 0.05 for all) ranging from 0.401 to 0.714. No significant correlation was found in ΔMK-SLG-R%, ΔMD-SLG-L%, and ΔMK-SLG-L% (*p* > 0.05) (detailed information seen in **Table 3**, **Figure 6A**).

**Table 3:** Correlation analysis of the change ratio of the DKI metrics of the salivary glands from pre-RT to post-RT with the dose of ipsilateral salivary glands.

	*r*	*p*
ΔMD-PG-R%	0.605	**0.000**
ΔMK-PG-R%	0.616	**0.000**
ΔMD-PG-L%	0.714	**0.000**
ΔMK-PG-L%	0.349	**0.023**
ΔMD-SMG-R%	0.401	**0.008**
ΔMK-SMG-R%	0.378	**0.013**
ΔMD-SMG-L%	0.596	**0.000**
ΔMK-SMG-L%	0.665	**0.000**
ΔMD-SLG-R%	0.485	**0.001**
ΔMK-SLG-R%	0.096	0.544
ΔMD-SLG-L%	0.256	0.097
ΔMK-SLG-L%	0.126	0.425

r represents correlation coefficients. The correlation analysis was conducted between the change ratio of the DKI parameters (MD and MK) from pre-RT to post-RT with the dose of ipsilateral salivary glands. The bold values represent p<0.05. Abbreviations are referred to in **Table 2**.

### Correlations Between the Change Ratio of the DKI Metrics With the Change Ratio of uSFR, sSFR, and XQ Scores From Pre-RT to Post-RT

ΔuSFR% was correlated with ΔMD-SMG% (*p* = 0.01), ΔMD-SLG% (*p* < 0.001), and ΔMK-SMG% (*p* < 0.001), with correlation coefficients of −0.39, −0.532, and 0.493 respectively. ΔsSFR% was correlated with ΔMD-PG% (*p* = 0.001), ΔMD-SMG% (*p* = 0.015), and ΔMK-PG% (*p* < 0.001), with correlation coefficients of −0.509, −0.221, and 0.524, respectively. ΔXQ% was only correlated with ΔMK-PG% (*p* = 0.004), with correlation coefficients of 0.433 (detailed information seen in **Table 4**, **Figure 6B**).

**Table 4:** Correlation analysis of the change ratio of the DKI metrics with the change ratio of uSFR, sSFR, and XQ scores from pre-RT to post-RT.

	ΔuSFR%, *r* (*p*)	ΔsSFR%, *r* (*p*)	ΔXQ%, *r* (*p*)
ΔMD-PG%	−0.180 (0.253)	**−0.509 (0.001)**	0.253 (0.106)
ΔMD-SMG%	**−0.39 (0.010)**	**−0.221 (0.015)**	**0.346 (0.024)**
ΔMD-SLG%	**−0.532 (0.000)**	−0.09 (0.547)	0.137 (0.386)
ΔMK-PG%	0.105 (0.469)	**0.524 (0.000)**	**−0.433 (0.004)**
ΔMK-SMG%	**0.493 (0.000)**	0.219 (0.163)	−0.192 (0.223)
ΔMK-SLG%	0.167 (0.290)	0.017 (0.913)	−0.058 (0.713)

r represents correlation coefficient. The bold values represent p<0.05.

## Discussion

This prospective study characterized the changing trend of MD and MK in bilateral major salivary glands, uSFR, sSFR, and XQ scores at the time of pre-IC, pre-RT, mid-RT, post-RT, 1m-RT, and 3m-RT. The most important finding of the present study lay in the potential value in tracking and monitoring the change of salivary glands during treatment and follow-up periods. The change ratio of DKI metrics (MD/MK) from pre-RT to post-RT was significantly correlated with the dose of bilateral salivary glands, and the change ratio of uSFR, sSFR, and XQ scores. In addition, we also confirmed an interesting result that had never been reported before that IC does have influences on salivary glands from the perspective of imaging in NPC.

The present study reconfirmed that there were no significant differences in MD and MK between the right and left sides of PG, SMG, and SLG. This finding was in accordance with previous investigations in terms of ADC (10, 22). In general, MD was significantly higher in SMG and SLG than that in PG, which was also in line with previous studies (6, 10, 22). It is thought to reflect the hallmark of the lower proportional amount of exocellular water (22) or higher fat content of PGs (6). As MK value reflects tissue structural complexity to some extent (20), less structural complexity induces less non-Gaussian water molecular diffusion, resulting in a lower MK. In our study, the MK was lower in SMG and SLG than PG, which might be ascribed to the different acinar cells in these glands. PG is composed of serous acini while SMG and SLG are mixed glands containing both mucous and serous acini (23). These assumptions also corresponded to the results that there were no differences in MD/MK between SMG and SLG in the study. Additionally, we also noticed that sSFR was significantly higher than uSFR at each time point in line with the previous studies (10).

After two cycles of IC, significant differences were detected in MD and MK of bilateral SMG and MD of SLG. Meanwhile, MD tends to increase over time, while the tendency of MK was reversed. These results were supported by a study reported by Jensen et al. who indicated that the acinar and ductal cell functions could be affected by adjuvant chemotherapy in breast cancer (11). However, our study showed no differences in uSFR, sSFR, and XQ after IC. A potential explanation for the conflicting results of SFR/XQ and DKI might be that the sensitivity of SFR or XQ is worse than that of DKI in detecting microstructural changes of the acinar cells. The research failed to detect a significant difference in MK of SLG before and after IC, which was most likely because MK may be sensitive to tumor tissue but seems to be less sensitive to normal structures like the salivary glands. Additionally, the tiny size of the sublingual gland might have biased the ultimate result caused by measurement error. Generally, we believe that IC indeed has an adverse effect on the salivary glands in patients with nasopharyngeal cancer. However, similar findings have not been documented to our knowledge.

The study found that MD of bilateral PG, SMG, and SLG at mid-RT (t3) and post-RT (t4) significantly increased while MK decreased compared with that of pre-RT, and the comparison between mid- and post-RT was also significant. The trend of MD is in accord with the change in ADC that had been reported in a previous study (10), which also corroborates the research on evaluation of RT-induced salivary gland damage (21, 24, 25). The increase in MD suggested increased water diffusivity as a result of RT-induced apoptosis of acinar cells and decrease in tissue cellular packing density (6, 10, 22) while reduced MK depends on the structural complexity of the glands (20, 21). Decreased diffusion kurtosis together with increased mean diffusivity may indicate increased extracellular space within the voxel of interest. The values of uSFR and sSFR decreased while XQ increased distinctly, which agreed with evidence from clinical observation (26), providing more corroboration of these results in terms of salivary function. We found that the change ratio of MD increased while the change ratio of MK decreased with the increasing dose of bilateral PG, SMG, and SLG. Furthermore, moderate-strong correlations were detected in the change ratio of the DKI metrics from pre-RT to post-RT with the dose of PG and SMG. The correlation between DKI parameters and dose of salivary glands in our subjects suggested that the changes in the microstructure detected by DKI corresponded to the radiation-induced damage of salivary glands. Our study also indicated that ΔuSFR% was correlated with ΔMD-SMG%, ΔMD-SLG%, and ΔMK-SMG%, while ΔsSFR% was correlated with ΔMD-PG%, ΔMD-SMG%, and ΔMK-PG%. These results implied that the change ratio of uSFR tend to be more related to SMG and SLG while sSFR tend to be related to PG. This could be explained by the fact that SMG and SLG mainly produce saliva under resting conditions (27). ΔXQ% was only correlated with ΔMK-PG%, which might be explained by the low reproducibility and non-specificity of XQ.

Compared with post-RT (t4), a significant decrease was found in sSFR at 1m-RT(t5), while uSFR and XQ scores showed no significant differences. The stable uSFR from post-RT to 1m-RT indicated that salivary gland function was conserved in the resting state. A potential explanation for this was that sparing the SMG technique for patients in our study contributed to less damage to SMG. PG produces approximately 50% of the total volume of stimulated whole saliva while the majority of saliva is secreted by SMG under resting conditions (9). Thus, we inferred that SMG is strongly linked to the uSFR, while PG is more likely to correlate with sSFR. There were no differences between 1m- and 3m-RT in our study. We speculated that radiation-induced acinar cellular loss, together with partial functional recovery ascribed to progenitor cells in salivary glands (27), led to an equilibration period for xerostomia in general.

The strengths of this study are its prospective design with identical treatment regimens, increased dependability of the research process, and the credibility of the results. Furthermore, this is the first try to track the dynamic change of the major salivary glands by DKI from pre-IC to 3m-RT. Lastly, IC-induced change of salivary glands was verified by DKI for the first time in patients with NPC. However, the present study is not without its limitations. First of all, the relatively small sample size constrained the generalizability of the results to some extent, and our findings need to be verified by a larger sample size study. Secondly, although this research was aimed to evaluate acute xerostomia within 3 months after RT, late xerostomia is even more important for NPC patients. Murthy et al. suggested that SMG function declines after IMRT with a nadir at 12 months and there is incomplete recovery over time with continued improvement over 24 months (28). Thus, an extension of follow-up periods to 24 months is necessary to identify salivary glands’ hypofunction recovery. Last but not least, the stem/progenitor cells in the parotid gland, which are of capacity to regenerate, are more sensitive to ionizing radiation (27). Thus, dividing salivary glands into radio-sensitive and insensitive zones according to the distribution of stem/progenitor cells for measurement of MD and MK is believed to be more accurate in evaluating salivary gland damage.

## Conclusion

Our study showed that MD of PG was lower while MK was higher than that of ipsilateral SMG and SLG at baseline. No significant differences were detected in MD/MK of ipsilateral SMG and SLG. The sSFR was significantly higher than the uSFR at each time point. MD of the salivary glands and XQ scores increased while MK, uSFR, and sSFR decreased significantly after IC+CCRT. The change ratio of DKI metrics (MD/MK) was significantly correlated with the dose of bilateral salivary glands and the change ratio of uSFR, sSFR, and XQ scores. DKI is a promising tool for tracking and monitoring the acute damage of PG, SMG, and SLG induced by IC+CCRT in NPC patients.

## Data Availability Statement

The raw data supporting the conclusions of this article will be made available by the authors, without undue reservation.

## Ethics Statement

This study was approved by the ethics board of the Chinese PLA General Hospital, and registered on July 6, 2019, in the Chinese Clinical Trial Registry (ChiCTR1900024328) (http://www.chictr.org.cn/showproj.aspx?proj=40726). Written informed consents were obtained before enrollment. The patients/participants provided their written informed consent to participate in this study.

## Author Contributions

Conception and design of the study: D-WZ, X-MF, S-HZ, W-JF, and LM. Data curation: D-WZ, X-MF, S-HZ, LM, Y-R L, JW, KL, LLM, GL, J-FL, XZ, ML, X-FQ, W-JF, and X-XZ. Data analyses: D-WZ, X-MF, S-HZ, LM, H-YJ. Drafting of the manuscript or revising it for important intellectual content: D-WZ, X-MF, LM, and X-XZ. All authors contributed to the article and approved the submitted version.

## Conflict of Interest

The authors declare that the research was conducted in the absence of any commercial or financial relationships that could be construed as a potential conflict of interest.

## Publisher’s Note

All claims expressed in this article are solely those of the authors and do not necessarily represent those of their affiliated organizations, or those of the publisher, the editors and the reviewers. Any product that may be evaluated in this article, or claim that may be made by its manufacturer, is not guaranteed or endorsed by the publisher.

## References

[1] Chen Y-P Chan ATC Le Q-T Blanchard P Sun Y Ma J. Nasopharyngeal Carcinoma. Lancet (2019) 394(10192):64–80. doi: 10.1016/s0140-6736(19)30956-0 31178151

[2] Wang K Tepper JE. Radiation Therapy-Associated Toxicity: Etiology, Management, and Prevention. CA Cancer J Clin (2021) 71(5):437–54. doi: 10.3322/caac.21689 34255347

[3] Yang H Chen X Lin S Rong J Yang M Wen Q. Treatment Outcomes After Reduction of the Target Volume of Intensity-Modulated Radiotherapy Following Induction Chemotherapy in Patients With Locoregionally Advanced Nasopharyngeal Carcinoma: A Prospective, Multi-Center, Randomized Clinical Trial. Radiother Oncol (2018) 126(1):37–42. doi: 10.1016/j.radonc.2017.07.020 28864073

[4] Liu LT Chen QY Tang LQ Guo SS Guo L Mo HY. Neoadjuvant or Adjuvant Chemotherapy Plus Concurrent CRT Versus Concurrent CRT Alone in the Treatment of Nasopharyngeal Carcinoma: A Study Based on EBV DNA. J Natl Compr Canc Netw (2019) 17(6):703–10. doi: 10.6004/jnccn.2018.7270 31200353

[5] Kong L Zhang Y Hu C Guo Y Lu JJ. Effects of Induction Docetaxel, Platinum, and Fluorouracil Chemotherapy in Patients With Stage III or IVA/B Nasopharyngeal Cancer Treated With Concurrent Chemoradiation Therapy: Final Results of 2 Parallel Phase 2 Clinical Trials. Cancer (2017) 123(12):2258–67. doi: 10.1002/cncr.30566 28192641

[6] Fan WJ Teng F Liu G Zhao DW Li JF Luo YR. Diffusion Weighted Imaging in Submandibular Gland Sparing Helical Tomotherapy for Nasopharyngeal Carcinoma. Radiother Oncol (2021) 157:247–54. doi: 10.1016/j.radonc.2021.02.004 33587972

[7] Saiki JP Cao H Van Wassenhove LD Viswanathan V Bloomstein J Nambiar DK. Aldehyde Dehydrogenase 3A1 Activation Prevents Radiation-Induced Xerostomia by Protecting Salivary Stem Cells From Toxic Aldehydes. Proc Natl Acad Sci U S A (2018) 115(24):6279–84. doi: 10.1073/pnas.1802184115 PMC6004437 29794221

[8] Pan XB Liu Y Li L Qu S Chen L Liang SX. Prognostic Nomogram of Xerostomia for Patients With Nasopharyngeal Carcinoma After Intensity-Modulated Radiotherapy. Aging (Albany NY) (2020) 12(2):1857–66. doi: 10.18632/aging.102717 PMC7053606 32005029

[9] Mercadante V Jensen SB Smith DK Bohlke K Bauman J Brennan MT. Salivary Gland Hypofunction and/or Xerostomia Induced by Nonsurgical Cancer Therapies: ISOO/MASCC/ASCO Guideline. J Clin Oncol (2021) 39(25):2825–43. doi: 10.1200/JCO.21.01208 34283635

[10] Fan WJ Teng F Luo YR Yu W Zhang Q Lu YP. Diffusion-Weighted Imaging as a Follow-Up Modality for Evaluation of Major Salivary Gland Function in Nasopharyngeal Carcinoma Patients: A Preliminary Study. Strahlenther Onkol (2020) 196(6):530–41. doi: 10.1007/s00066-020-01580-5 PMC7248033 32025803

[11] Jensen SB Mouridsen HT Reibel J Brunner N Nauntofte B. Adjuvant Chemotherapy in Breast Cancer Patients Induces Temporary Salivary Gland Hypofunction. Oral Oncol (2008) 44(2):162–73. doi: 10.1016/j.oraloncology.2007.01.015 17588802

[12] Garcia-Chias B Figuero E Castelo-Fernandez B Cebrian-Carretero JL Cerero-Lapiedra R. Prevalence of Oral Side Effects of Chemotherapy and its Relationship With Periodontal Risk: A Cross Sectional Study. Sup Care Cancer (2019) 27(9):3479–90. doi: 10.1007/s00520-019-4650-6 30675665

[13] Jardim LC Flores PT do Carmo Dos Santos Araujo M Chiesa J de Moraes CMB Antoniazzi RP. Oral Health-Related Quality of Life in Breast Cancer Survivors. Sup Care Cancer (2020) 28(1):65–71. doi: 10.1007/s00520-019-04792-3 30982094

[14] Jensen SB Pedersen AM Vissink A Andersen E Brown CG Davies AN. A Systematic Review of Salivary Gland Hypofunction and Xerostomia Induced by Cancer Therapies: Management Strategies and Economic Impact. Sup Care Cancer (2010) 18(8):1061–79. doi: 10.1007/s00520-010-0837-6 20333412

[15] Barazzuol L Coppes RP van Luijk P. Prevention and Treatment of Radiotherapy-Induced Side Effects. Mol Oncol (2020) 14(7):1538–54. doi: 10.1002/1878-0261.12750 PMC7332214 32521079

[16] Hawkins PG Lee JY Mao Y Li P Green M Worden FP. Sparing All Salivary Glands With IMRT for Head and Neck Cancer: Longitudinal Study of Patient-Reported Xerostomia and Head-and-Neck Quality of Life. Radiother Oncol (2018) 126(1):68–74. doi: 10.1016/j.radonc.2017.08.002 28823405

[17] Tenhunen M Collan J Kouri M Kangasmaki A Heikkonen J Kairemo K. Scintigraphy in Prediction of the Salivary Gland Function After Gland-Sparing Intensity Modulated Radiation Therapy for Head and Neck Cancer. Radiother Oncol (2008) 87(2):260–7. doi: 10.1016/j.radonc.2008.02.017 18329118

[18] Chen W Su GY Zhou Y Jiang JS Jiang RH Bao ML. Longitudinal Multiparametric MRI Assessment of Irradiated Salivary Gland in a Rat Model: Correlated With Histological Findings. J. Magn Reson Imaging (2021) 54(6):1730–41. doi: 10.1002/jmri.27836 34278649

[19] Teng F Fan W Luo Y Ju Z Gong H Ge R. Reducing Xerostomia by Comprehensive Protection of Salivary Glands in Intensity-Modulated Radiation Therapy With Helical Tomotherapy Technique for Head-And-Neck Cancer Patients: A Prospective Observational Study. BioMed Res Int (2019) 2019:2401743. doi: 10.1155/2019/2401743 31380414 PMC6662416

[20] Rosenkrantz AB Padhani AR Chenevert TL Koh DM De Keyzer F Taouli B. Body Diffusion Kurtosis Imaging: Basic Principles, Applications, and Considerations for Clinical Practice. J. Magn Reson Imaging (2015) 42(5):1190–202. doi: 10.1002/jmri.24985 26119267

[21] Zhou N Chen W Pan X He J Yan J Zhou Z. Early Evaluation of Radiation-Induced Parotid Damage With Diffusion Kurtosis Imaging: A Preliminary Study. Acta Radiol (2018) 59(2):212–20. doi: 10.1177/0284185117710051 28509567

[22] Loimu V Seppala T Kapanen M Tuomikoski L Nurmi H Makitie A. Diffusion-Weighted Magnetic Resonance Imaging for Evaluation of Salivary Gland Function in Head and Neck Cancer Patients Treated With Intensity-Modulated Radiotherapy. Radiother Oncol (2017) 122(2):178–84. doi: 10.1016/j.radonc.2016.07.008 27475276

[23] Holmberg KV Hoffman MP. Anatomy, Biogenesis and Regeneration of Salivary Glands. Monogr Oral Sci (2014) 24:1–13. doi: 10.1159/000358776 24862590 PMC4048853

[24] Shi D Qian JJ Fan GH Shen JK Tian Y Xu L. Salivary Gland Function in Nasopharyngeal Carcinoma Before and Late After Intensity-Modulated Radiotherapy Evaluated by Dynamic Diffusion-Weighted MR Imaging With Gustatory Stimulation. BMC Oral Health (2019) 19(1):288. doi: 10.1186/s12903-019-0951-x 31864328 PMC6925496

[25] Zhou N Chu C Dou X Li M Liu S Zhu L. Early Evaluation of Irradiated Parotid Glands With Intravoxel Incoherent Motion MR Imaging: Correlation With Dynamic Contrast-Enhanced MR Imaging. BMC Cancer (2016) 16(1):865. doi: 10.1186/s12885-016-2900-2 27821130 PMC5100256

[26] Lan X Chan JYK Pu JJ Qiao W Pang S Yang WF. Saliva Electrolyte Analysis and Xerostomia-Related Quality of Life in Nasopharyngeal Carcinoma Patients Following Intensity-Modulated Radiation Therapy. Radiother Oncol (2020) 150:97–103. doi: 10.1016/j.radonc.2020.06.016 32544605

[27] van Luijk P Pringle S Deasy JO Moiseenko VV Faber H Hovan A. Sparing the Region of the Salivary Gland Containing Stem Cells Preserves Saliva Production After Radiotherapy for Head and Neck Cancer. Sci. Transl Med (2015) 7(305):305ra147. doi: 10.1126/scitranslmed.aac4441 PMC4964284 26378247

[28] Murthy V Lewis S Kannan S Khadanga CR Rangarajan V Joshi K. Submandibular Function Recovery After IMRT in Head and Neck Cancer: A Prospective Dose Modelling Study. Radiother Oncol (2018) 129(1):38–43. doi: 10.1016/j.radonc.2018.02.021 29724411

