# Supplementary material for: Application of Diffusion Kurtosis Imaging in Evaluating Acute Xerostomia in Nasopharyngeal Carcinoma Treated With Induction Chemotherapy Plus Concurrent Chemoradiotherapy
Source: Front Oncol. 2022 May 19;12:870315. doi: 10.3389/fonc.2022.870315 (PMC9162117; doi:10.3389/fonc.2022.870315)
Supplement: Supplementary file 1 [file DataSheet_1.pdf]

Supplementary Material

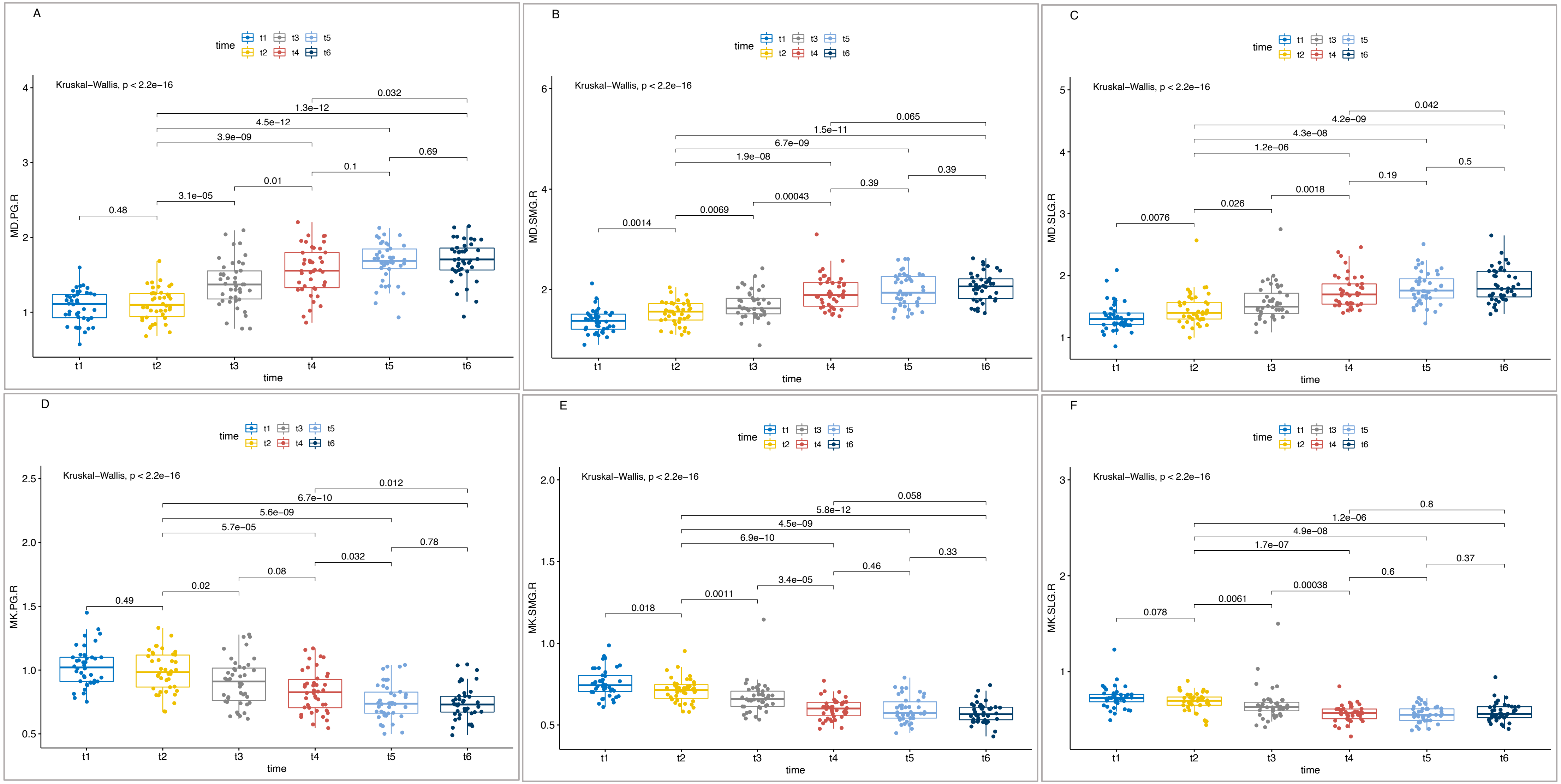

Fig.7 A~F the pairwise comparisons of MD and MK of right PG, SMG, between different time points, p<0.05 denotes significant differences between two time points linked together. MD: mean diffusion; MK: mean kurtosis. PG: parotid gland; SMG: submandibular gland; SLG: sublingual gland. time: t1:prior to induction chemotherapy; t2: prior to radiotherapy; t3: middle of radiotherapy; t4: immediately after radiotherapy; t5: 1 months after radiotherapy; t6: 3 months after radiotherapy.
